# Supplementary material for: Knowledge, attitude, and practice of the National Guidelines for Diagnosis and Treatment of Malaria among medical doctors in Ebonyi state, Nigeria: A cross-sectional survey
Source: PLoS One. 2021 Sep 20;16(9):e0257600. doi: 10.1371/journal.pone.0257600 (PMC8451992; doi:10.1371/journal.pone.0257600)
Supplement: S1 Questionnaire — (DOCX) [file pone.0257600.s001.docx]

**MEDICAL DOCTORS’ QUESTIONNAIRE**

**ON**

***Knowledge, Attitude, and Practice of the National Guidelines for Diagnosis and Treatment of Malaria***

Greetings. My name is Omale Ugwu Innocent. I am doing a Certificate Course in Health Policy and Health Systems in Ebonyi State University. I am carrying out a research (survey) on the above subject among medical doctors in Ebonyi State. The research was granted ethical approval by the Research and Ethics Committee (REC) of Ebonyi State University. The questionnaire usually takes about 10 minutes to complete. Your consent to participate in the survey is completely voluntary. Your participation in the survey will be very much appreciated. Any information you provide will be protected strictly from other persons. **NOTE:** Only medical doctors that are involved in management of malaria patients should fill the questionnaire.

**SECTION 1: RESPONDENT’S BACKGROUND**

| **S/N** | **QUESTIONS** | **RESPONSE** **(CIRCLE)** |
| --- | --- | --- |
| 1. | Age as at last birth day? | <= 30 ………………………………………….. 1  31-40 …………………………………………. 2  41-50 ………………………………………….. 3  > 50 …………………………………………… 4 |
| 2. | Sex | MALE ……………………….................. 1  FEMALE ……………………………………….. 2 |
| 3. | Marital status? | MARRIED …………………………………….. 1  DIVORCED/SEPARATED ……………….. 2  WIDOWED …………………………………… 3  SINGLE…………………………………………. 4 |
| 4. | Professional rank? | HOUSE OFFICER ………………………….. 1  REGISTRAR ………………………………….. 2  SENIOR REGISTRAR ……………………… 3  MEDICAL OFFICER ……………………….. 4  CONSULTANT ………………………......... 5 |
| 5. | Years of experience (duration of practice)? | <= 5 YEARS ………………………............. 1  6-10 YEARS ………………………………….. 2  11-15 YEARS ………………………………… 3  16-20 YEARS ………………………………… 4  > 20 YEARS ………………………………….. 5 |
| 6. | Current primary place of practice? | PRIVATE HOSPITAL ………………………… 1  MISSIONARY HOSPITAL …………………. 2  GENERAL HOSPITAL ……………………….. 3  AEFUTHA ………………………………………. 4  OTHER …6  SPECIFY |
| 7. | **IF YOU PRACTICE IN AEFUTHA:**  What is your department? | COMMUNITY MEDICINE ………………… 1  FAMILY MEDICINE…………………………… 2  INTERNAL MEDICINE ……………………… 3  OBS & GYNAE ………………………………… 4  PAEDIATRICS ……………………………………5  OTHER …6  SPECIFY |

**SECTION 2: RESPONDENT’S KNOWLEDGE OF THE GUIDELINE**

| 8. | Are you aware there is a national guideline for diagnosis and treatment of malaria? | YES ………………………........ 1  NO ……………………………… 2 SKIP  **12**  TO |
| --- | --- | --- |
| 9. | Have you seen the current (2015) guideline? | YES ………………………........ 1  NO ……………………………… 2 SKIP  **11**  TO |
| 10. | Have you read the current (2015) guideline? | YES ………………………............................. 1  NO ……………………………………………………. 2 |
| 11. | What are your sources of information about the current guideline?  **CIRCLE ALL THAT APPLY** | I HAVE THE GUIDELINE ……………………… 1  COLLEAGUES ……………………………………. 2  SEMINARS/CLINICAL MEETINGS ………… 3  TRAINING/CME …………………………………. 4  OTHER …….6  SPECIFY |
| 12. | It is recommended that all patients suspected of having uncomplicated malaria receive treatment based on confirmation of diagnosis with? | MICROSCOPY …………………………………….. 1  MALARIA RDT …………………………………… 2  MICROSCOPY OR MALARIA RDT ………… 3  CLINICAL SUSPICION …………………………. 4  DON’T KNOW …………………………………… 8 |
| 13. | It is recommended that the result of parasitological diagnosis (with microscopy or malaria RDT (MRDT)) of uncomplicated malaria be available within how many hours of patients’ presentation? | HALF AN HOUR ………………………............ 1  TWO HOURS …………………………………….. 2  SIX HOURS ………………………………………… 3  EIGHT HOURS …………………………………… 4  TWENTY FOUR HOURS ………………………. 5  DON’T KNOW …………………………………… 8 |
| 14. | Anti-malarial drugs should be limited to? | ONLY TEST POSITIVE CASES ………………. 1  TEST POSITIVE & SOME TEST NEGATIVE CASES ………………………. 2  DON’T KNOW ……………………………………. 8  { |
| 15. | Is parallel (simultaneous) testing with microscopy and MRDT recommended for diagnosing uncomplicated malaria? | YES ………………………............................. 1  NO ……………………………………………………. 2  DON’T KNOW …………………………………… 8 |
| 16. | Treatment based solely on clinical suspicion should only be considered in under-5 children when? | MICROSCOPY IS NOT ACCESSIBLE ……… 1  MALARIA RDT IS NOT ACCESSIBLE ……. 2  MICROSCOPY OR MALARIA RDT IS NOT ACCESSIBLE …… 3  DON’T KNOW …………………………………… 8 |
| 17. | What is the recommended anti-malarial drug for uncomplicated malaria? | SP/FANSIDAR/MALOXINE/AMALAR …...1  CHLOROQUINE …………………………......... 2  QUININE ………………………………………….. 3  ACTs …………………………………………………. 4  OTHER …….6  SPECIFY |
| 18. | **IF NO. 17 ABOVE IS ACTs:**  What is the ACT of choice (first choice)? | ARTEMETHER-LUMEFANTRINE …………. 1  ARTESUNATE-AMODIAQUINE …………… 2  ARTESUNATE-MEFLOQUINE …………… 3  DIHYDROARTEMISININ-PIPERAQUIN … 4  ARTEMISININ-PIPERAQUIN ……………….. 5  OTHER …….6  SPECIFY |
| 19. | **IF NO. 17 ABOVE IS ACTs:**  What is the alternate ACT (second choice)? | ARTEMETHER-LUMEFANTRINE …………. 1  ARTESUNATE-AMODIAQUINE …………… 2  ARTESUNATE-MEFLOQUINE …………… 3  DIHYDROARTEMISININ-PIPERAQUIN … 4  ARTEMISININ-PIPERAQUIN ……………….. 5  OTHER …….6  SPECIFY |
| 20. | What is the recommended anti-malarial drug for lactating mothers with uncomplicated malaria? | SP/FANSIDAR/MALOXINE/AMALAR …...1  CHLOROQUINE …………………………......... 2  QUININE ………………………………………….. 3  ACTs …………………………………………………. 4  OTHER …….6  SPECIFY |
| 21. | What is the recommended anti-malarial drug for uncomplicated malaria in **second and third trimesters** of pregnancy? | SP/FANSIDAR/MALOXINE/AMALAR …...1  CHLOROQUINE …………………………......... 2  QUININE ………………………………………….. 3  ACTs …………………………………………………. 4  OTHER …….6  SPECIFY |
| 22. | What is the recommended anti-malarial drug for uncomplicated malaria in **first trimester** of pregnancy? | SP/FANSIDAR/MALOXINE/AMALAR …...1  CHLOROQUINE …………………………......... 2  QUININE + CLINDAMYCIN (FOR 7 DAYS) …………………………………….. 3  ACTs ………………………………………………….. 4  OTHER …….6  SPECIFY |
| 23. | What is the alternate anti-malarial drug **(second choice)** for uncomplicated malaria in **first trimester** of pregnancy? | SP/FANSIDAR/MALOXINE/AMALAR …...1  CHLOROQUINE …………………………......... 2  QUININE + CLINDAMYCIN (FOR 7 DAYS) …………………………………….. 3  ACTs ………………………………………………….. 4  OTHER …….6  SPECIFY |
| 24. | Should all suspected cases of severe malaria receive parasitological diagnosis (with microscopy or MRDT) before treatment? | YES ………………………............................. 1  NO ……………………………………………………. 2  DON’T KNOW ………………………………………8 |
| 25. | What is the recommended anti-malarial drug for severe malaria? | IV/IM ARTESUNATE …………………………… 1  IV/IM ARTEMETHER …………………………… 2  IV/IM QUININE …………………………………….3  ACTs …………………………………………………… 4  OTHER …… 6  SPECIFY |
| 26. | What is (are) the alternate anti-malarial drug(s) (**second choice**) for severe malaria?  **CIRCLE ALL THAT APPLY** | IV/IM ARTESUNATE ………………………………1  IV/IM ARTEMETHER …………………………… 2  IV/IM QUININE …………………………………….3  ACTs …………………………………………………… 4  OTHER ………6  SPECIFY |
| 27. | Once started, IV/IM anti-malarial for severe malaria should be given for a minimum of what? | 12 HOURS …………………………………………… 1  24 HOURS …………………………………………… 2  48 HOURS …………………………………………… 3  72 HOURS …………………………………………… 4  DON’T KNOW ……………………………………… 8 |
| 28. | After IV/IM anti-malarial for severe malaria, should the patient be given the full dose of an ACT? | YES ……………………….............................. 1  NO ……………………………………………………… 2  DON’T KNOW …………………………………… 8 |
| 29. | What is the recommended anti-malarial drug for severe malaria in **second and third trimesters** of pregnancy? | IV/IM ARTESUNATE ……………………………. 1  IV/IM ARTEMETHER …………………………… 2  IV/IM QUININE …………………………………… 3  ACTs …………………………………………………… 4  OTHER ………6  SPECIFY |
| 30. | What is the recommended anti-malarial drug for severe malaria in **first trimester** of pregnancy? | IV/IM ARTESUNATE ……………………………. 1  IV/IM ARTEMETHER ……………………………. 2  IV/IM QUININE ……………………………………. 3  ACTs ……………………………………………………. 4  OTHER …….. 6  SPECIFY |

**SECTION 3: RESPONDENT’S ATTITUDE TOWARDS THE GUIDELINE**

|  | **Please CIRCLE one of the options from 1-5 for each of the statements below** | **STRONGLY DIASGREE** | **DISAGREE** | **UNDECIDED/UNSURE** | **AGREE** | **STRONGLY AGREE** |
| --- | --- | --- | --- | --- | --- | --- |
| 31. | All suspected cases of uncomplicated malaria should receive treatment based on parasitological diagnosis (with microscopy or malaria RDT (MRDT)) | 1 | 2 | 3 | 4 | 5 |
| 32. | Only patients with positive (microscopy or MRDT) test results should be given anti-malarial drugs | 1 | 2 | 3 | 4 | 5 |
| 33. | Patients with negative microscopy results should not be given anti-malarial drugs | 1 | 2 | 3 | 4 | 5 |
| 34. | Patients with negative MRDT results should not be given anti-malarial drugs | 1 | 2 | 3 | 4 | 5 |
| 35. | Treatment based solely on clinical features should only be considered in under-5 children when microscopy or MRDT is not accessible | 1 | 2 | 3 | 4 | 5 |
| 36. | The anti-malarial drug of choice is ACTs | 1 | 2 | 3 | 4 | 5 |
| 37. | The ACT of choice is artemether-lumefantrine while artesunate-amodiaquine is the alternate ACT | 1 | 2 | 3 | 4 | 5 |
| 38. | All suspected cases of severe malaria should receive parasitological diagnosis (with microscopy or MRDT) before treatment | 1 | 2 | 3 | 4 | 5 |
| 39. | IV/IM anti-malarial for severe malaria should be given for a minimum of 24 hours once it is commenced | 1 | 2 | 3 | 4 | 5 |
| 40. | After IV/IM anti-malarial for severe malaria the patient should be given the full dose of an ACT | 1 | 2 | 3 | 4 | 5 |
| 41. | SP/fansidar/maloxine/amalar should only be used for IPT in pregnancy (not to treat malaria) | 1 | 2 | 3 | 4 | 5 |
| 42. | Chloroquine should not be used to treat malaria | 1 | 2 | 3 | 4 | 5 |

**SECTION 4: RESPONDENT’S PRACTICE OF THE GUIDELINE**

|  | **How frequently do you do the following?**  **Please CIRCLE one option** | **NEVER** | **RARELY** | **SOMETIMES** | **OFTEN** | **ALWAYS** |
| --- | --- | --- | --- | --- | --- | --- |
| 43. | Use only clinical features (presumptive diagnosis) as the basis for treating uncomplicated malaria | 1 | 2 | 3 | 4 | 5 |
| 44. | Prescribe anti-malarial drugs to patients with positive microscopy test results | 1 | 2 | 3 | 4 | 5 |
| 45. | Prescribe anti-malarial drugs to patients with positive malaria RDT (MRDT) test results | 1 | 2 | 3 | 4 | 5 |
| 46. | Prescribe anti-malarial drugs to patients with negative microscopy test results | 1 | 2 | 3 | 4 | 5 |
| 47. | Prescribe anti-malarial drugs to patients with negative MRDT test results | 1 | 2 | 3 | 4 | 5 |
| 48. | Use ACTs to treat uncomplicated malaria | 1 | 2 | 3 | 4 | 5 |
| 49. | Use artemether-lumefantrine to treat uncomplicated malaria | 1 | 2 | 3 | 4 | 5 |
| 50. | Use artesunate-amodiaquine to treat uncomplicated malaria | 1 | 2 | 3 | 4 | 5 |
| 51. | Use only clinical features (presumptive diagnosis) as the basis for treating severe malaria | 1 | 2 | 3 | 4 | 5 |
| 52. | Prescribe IV/IM anti-malarial for severe malaria | 1 | 2 | 3 | 4 | 5 |
| 53. | Prescribe the full dose of an ACT after IV/IM anti-malarial for severe malaria | 1 | 2 | 3 | 4 | 5 |
| 54. | Prescribe SP/fansidar/maloxine/amalar for uncomplicated malaria | 1 | 2 | 3 | 4 | 5 |
| 55. | Prescribe chloroquine for uncomplicated malaria | 1 | 2 | 3 | 4 | 5 |
