# Supplementary material for: Knowledge, attitude, and practice of the National Guidelines for Diagnosis and Treatment of Malaria among medical doctors in Ebonyi state, Nigeria: A cross-sectional survey
Source: PLoS One. 2021 Sep 20;16(9):e0257600. doi: 10.1371/journal.pone.0257600 (PMC8451992; doi:10.1371/journal.pone.0257600)
Supplement: Attachment — Submitted filename: Review comments.docx [file pone.0257600.s003.docx]

**Review comments**

**General comment**

This is an interesting manuscript and well written. I commend the authors for a good work. Even though the sample size for the study is small, the relevance of the study cannot be in doubt.

Before this manuscript could be accepted for publication, Authors should explain the selection of the respondents. (Responses to the questions raised in this regard will be necessary). The authors should also take another look on the scoring of the attitude and practice variables or provide a reference to what they have done.

An explanation is also required on why all the variables merited inclusion in the multivariate logistic regression model.

**Abstract**

**Methods**

How were the medical doctors selected for inclusion in the study?

How many variables were used to assess the outcome variables?

Why categorize the outcome variables into three categories when authors had in mind to perform logistic regression analysis?

Authors should state the level of statistical significance.

**Results**

Authors should explain the meaning of adjusted p value

Present logistic regression analysis results using adjusted odds ratio and 95% confidence interval

The outcome variable should be well described. Is it good knowledge of what?

**Conclusions**

What are the public health implications of the study findings?

How could the attitude of the medical doctors towards the National Guideline be improved?

**Introduction**

My commendation to the authors for a well written introduction.

Line 82 appear like a stand-alone statement. To my knowledge…… This should be reviewed.

**Methods**

**Study design and setting**

The description of the study as ‘analytical cross-sectional study’ should be reviewed.

**Study participants and data collection**

The selection of the respondents is not very clear

Were all the medical doctors present on the day of data collection in the departments/units as stated in the manuscript included in the study?

Then if it was a total population study, what is the response rate.

The authors should give the readers an idea of the total number of doctors working in those areas where malaria cases are managed? As a follow up to that a good description of how the respondents were selected should be done.

Who designed the questionnaire and where was it pre-tested?

**Statistical analysis**

It will be good to state the number of variables used to assess the attitude of the respondents to the National Guideline. The scoring of the variables used to assess attitude of the respondents is not very clear. What is the rationale for awarding two marks to someone who is undecided on a particular variable? In any case, the authors should provide a reference that justifies this very decision.

Do the same for the scoring of the variables used to assess practice level of the respondents.

How was the practice level dichotomized?

**Results**

A proportion of 43% cannot be regarded as majority. Use highest proportion instead. Correct in other areas. Majority is recorded for proportions above 50%.

**Table 1**

Second column should be frequency (n=156)

Remove the row total (Do the same for other tables).

Figures 3A and 3B should be removed. Then for table 5, remove the crude odds ratios and replace with proportions that had good and poor practice. The p value for the bivariate analysis should be included. For the multivariate analysis, present the adjusted odds ratio and 95% confidence interval. The p value on multivariate analysis may be included.

Remove the constants in table 5

All the variables had both crude and adjusted odds ratios. What qualified the variables for inclusion in the multivariate logistic regression model? If all the variables merited inclusion as seen in the result, what is the rationale for that?

Considering the small sample size as seen in this study, why should duration of practice in years have 5 categories? This remarkably affected the adjusted odds ratio and should be reviewed. Review the number of categories for other independent variables.

**Discussion**

Include Nigeria after Ogun state for better understanding

Discuss the predictors of good practice of the National guideline

**Conclusions**

This should be in a single paragraph

Use predictor of good practice instead of strong predictor
