# Supplementary material for: Knowledge, attitude, and practice of the National Guidelines for Diagnosis and Treatment of Malaria among medical doctors in Ebonyi state, Nigeria: A cross-sectional survey
Source: PLoS One. 2021 Sep 20;16(9):e0257600. doi: 10.1371/journal.pone.0257600 (PMC8451992; doi:10.1371/journal.pone.0257600)
Supplement: Attachment — Submitted filename: Response to Reviewers.docx [file pone.0257600.s004.docx]

RESPONSE TO REVIEWERS

Reviewer # 1

Abstract

**Comment:** Line 32 - knowledge, attitude, and practice respectively for knowledge, attitude, and practice scores of <50% - delete the repeated words: knowledge, attitude, and practice and review the sentence;
**Response:** Please note that each respondent was categorized as having poor, fair, or good knowledge, attitude, and practice respectively **based on** the knowledge, attitude, and practice scores of <50%, 50–75%, and >75%. “for” has been replaced with “based on” for clarity and this might be more understandable than when the second “knowledge, attitude, and practice” is deleted viz: Each respondent was categorized as having poor, fair, or good knowledge, attitude, and practice respectively **for** scores of <50%, 50–75%, and >75%. In this case, the query that might arise is, “which scores?” So I think it should be indicated straightway that is was the knowledge, attitude, and practice scores.

**Comment:** Line 39 - Overall, 59 (37.8%) had good… Review. Change “Overall”.
**Response:** “Overall” have been replaced with “Among the medical doctors” in line 37 and removed from line 39.

**Comment:** Line 42 - predictor of good practice of the 2015 Guidelines (adjusted p-value of overall effect=0.0003). Eliminate the “of the 2015 Guidelines”.
**Response:** The phrase has been deleted.

Introduction

**Comments:**

Line 57: There were several recommendations in the 2015 guidelines. Eliminate the 2015.
Line 69: have the main responsibility for treating patients with both uncomplicated and severe malaria. Eliminate “both uncomplicated and severe”.
Line 71: knowledge of the guideline, have good attitude towards the guideline, and above all, have to practice. Eliminate the repeated verb, “have”.

**Response:** The corrections have been effected.

Methods

**Comments:**

Line 91: This study was an analytical cross-sectional study. The study was carried out in May, 2019 in Ebonyi state. Change to: “This study was an analytical cross-sectional survey carried out in May, 2019 in Ebonyi state” or other similar, to simplify the phrase.
Line 120 : Data was entered using Microsoft Excel 2007 (Microsoft Inc., Redmond, Washington), and was verified and… substitute the and was by a common .

**Response:** The corrections have been effected.

Results

**Comment:** Line 152: Majority of 108 (69.2%) were males and 48 (30.8%) were… Correct to Majority of them, 108 (69.2%) were males….
**Response:** The correction has been effected.

**Comment:** Line 155: and were in Community Medicine Department in the teaching hospital, 34 (21.8%) (Table 1). Change the order to: …and 34 (21.8%) were in Community…
**Response:** The starting phrase “The majority of the respondents:” applies to every other phrase in the compound sentence. Changing the order as suggested will alter the flow of the compound sentence.

**Comments:**

Page 9 - Line 185 to Line 201: Some phrases started by While… Please review it. May changing the word by other to give more sense to your results.
Line 222 : While 66 (42.3%) never, 52 (33.3%) rarely, 30 (19.2%) sometimes, 5 (3.2%) often, and 3 (1.9%) always prescribed… Change While… Start the phase to another word.
Line 226: Overall, while 59 (37.8%) had good practice of the 2015 National Guidelines for Diagnosis and Treatment of… Cut while.
Line 329: The fact that this study collected self-reported data about practices was a limitation because of due the possibility of… Change the word because of… It gives more sense.

**Response:** The corrections have been effected.

Reviewer # 2

Abstract
Methods

**Comments:**

How were the medical doctors selected for inclusion in the study?
How many variables were used to assess the outcome variables?

**Response:** They were selected by convenience sampling as stated in the body of the manuscript under the ”study participants and data collection” section. The numbers of variables used for the assessment have been added to the body of the manuscript. There is no space to include other details in the Abstract section due to the Journal’s word count.

**Comment:** Why categorize the outcome variables into three categories when authors had in mind to perform logistic regression analysis?
**Response:** Categorization the outcome variables into three categories are to convey more detailed information for the descriptive aspect of the analysis. This does not prevent dichotomization of the practice variable for inferential analysis.

**Comment:** Authors should state the level of statistical significance.
**Response:** The suggestion has been effected.

Results
**Comment:** Authors should explain the meaning of adjusted p value  **Response:** Just like adjusted odds ratio, adjusted p-value is p-value from adjusted analysis (multivariate analysis). It is not necessary to be explaining this in the manuscript.

**Comment:** Present logistic regression analysis results using adjusted odds ratio and 95% confidence interval
**Response:** For multi-level variable such as attitude level with three categories, the overall effect is preferably reported in the abstract. There is no space to present the pair-wise result as suggested.

**Comment:** The outcome variable should be well described. Is it good knowledge of what? **Response:** The outcome variables are knowledge, attitude, and practice.

Conclusions
**Comment:** What are the public health implications of the study findings?
**Response:** The implication of the main finding is as stated. The investigator cannot speculate on other implications.

**Comment:** How could the attitude of the medical doctors towards the National Guideline be improved?
**Response:** It could be through training and re-training of the medical doctors.

Introduction
**Comment:** Line 82 appear like a stand-alone statement. To my knowledge…… This should be reviewed.
**Response:** The investigator is the only author of the manuscript and seems not to understand this comment.

Methods
Study design and setting
**Comment:** The description of the study as ‘analytical cross-sectional study’ should be reviewed.
**Response:** The study was actually an analytical cross-sectional study

Study participants and data collection
**Comments:**
The selection of the respondents is not very clear
Were all the medical doctors present on the day of data collection in the departments/units as stated in the manuscript included in the study?
Then if it was a total population study, what is the response rate.
The authors should give the readers an idea of the total number of doctors working in those areas where malaria cases are managed? As a follow up to that a good description of how the respondents were selected should be done.

**Response:** Convenience sampling was employed as stated in the manuscript.

**Comment:** Who designed the questionnaire and where was it pre-tested?
**Response:** The questionnaire was designed by the investigator and pre-tested among doctors who were later not included in the study.

Statistical analysis
**Comments:**

It will be good to state the number of variables used to assess the attitude of the respondents to the National Guideline. The scoring of the variables used to assess attitude of the respondents is not very clear. What is the rationale for awarding two marks to someone who is undecided on a particular variable? In any case, the authors should provide a reference that justifies this very decision.
Do the same for the scoring of the variables used to assess practice level of the respondents.

**Response:** The numbers of variables have been added to the “study participants and data collection” section. The investigator believes of the scoring of attitude and practice is clearly described. Please note that, although many scholars might chose to respectively assign scores of 1–5 to the five responses, other scholars might chose to use different “weighted” scoring approach based on the concept of their study. For example, a study assigned scored of 1–4 to “strongly agree or disagree” or “somewhat agree or disagree” and “0” to “don’t know/can’t say” (Vaidya A, Aryal UR, Krettek A. Cardiovascular health knowledge, attitude and practice/behaviour in an urbanising community of Nepal: a population-based cross-sectional study from Jhaukhel-Duwakot Health Demographic Surveillance Site. BMJ Open 2013;3:e002976).

In this study, a participant who was “undecided” regarding a salient recommendation in a diagnosis and treatment guideline should receive a higher score than another who “disagreed” or “strongly disagreed”. However, the interval between scores is not a “mathematical derivation”.

Assigning the middle response with a score of zero, and positive and negative scores around the zero score, has also been suggested (<http://v2020eresource.org/content/files/guideline_kap_Jan_mar04.pdf>)

Scoring the most positive response option as 10, the least positive as zero, and the middle as 5 has also been recommended (<https://www.cqc.org.uk/sites/default/files/20151125_nhspatientsurveys_scoring_methodology.pdf>)

**Comment:** How was the practice level dichotomized?
**Response:** Practice level was dichotomized as good and fair or poor practice as shown in Figure 3A & B. The phrase has been added to the “statistical analysis” section.

Results
**Comment:** A proportion of 43% cannot be regarded as majority. Use highest proportion instead. Correct in other areas. Majority is recorded for proportions above 50%.
**Response:** The suggestion has been effected.

**Comments:**
Table 1
Second column should be frequency (n=156)
Remove the row total (Do the same for other tables).
Figures 3A and 3B should be removed. Then for table 5, remove the crude odds ratios and replace with proportions that had good and poor practice. The p value for the bivariate analysis should be included. For the multivariate analysis, present the adjusted odds ratio and 95% confidence interval. The p value on multivariate analysis may be included.
Remove the constants in table 5
All the variables had both crude and adjusted odds ratios. What qualified the variables for inclusion in the multivariate logistic regression model? If all the variables merited inclusion as seen in the result, what is the rationale for that?
Considering the small sample size as seen in this study, why should duration of practice in years have 5 categories? This remarkably affected the adjusted odds ratio and should be reviewed. Review the number of categories for other independent variables.

**Response:** The tables and figures are okay as presented and the style reflects the concept of the study. “n” connotes frequency and is the same as “f”. Some may chose to use “f”, others use “n”. Proportions are presented in figure 2 and must not be presented in table 5.

Please note that there are several criteria and methods for inclusion of variables in regression models and different scholars use different methods. The number of independent variables was modest so all were included in the model to minimize bias.

Discussion
**Comment:** Include Nigeria after Ogun state for better understanding
**Response:** The suggestion has been effected.

**Comment:** Discuss the predictors of good practice of the National guideline
**Response:** The investigator could not identify any similar studies that reported predictors of good practice for comparison and discussion.

Conclusions
**Comments:**
This should be in a single paragraph
Use predictor of good practice instead of strong predictor

**Response:** The suggestions have been effected.

Reviewer # 3

Title
**Comment:** The title does not match with the study objective. The title reflects a study which is purely descriptive, however, the objective of the study is to also assess factors associated with practices regarding the Malaria guideline. Kindly recast the title to reflect that the study assessed and reports about predictors.
**Response:** The title is okay as stated. There is nothing in the title that suggests a purely descriptive study. The basic design is included in the title, viz: “a cross-sectional survey”, which could be descriptive or analytical. Some titles of similar studies in the literature includes “predictors” or “associated factors” and others do not.

Introduction
**Comment:** Line 66: ….the risk of drug resistance. It should be “…………risk for development of drug resistance”
**Response:** The phrase is correct. If risk is the probability that an event will occur, then the phrase is okay.

**Comment:** Line 87-88: The aim of this study was to evaluate the knowledge, attitude, and practice of the 2015 National Guidelines for Diagnosis and Treatment of Malaria, and associated factors, among medical doctors in Ebonyi state, Nigeria. Rephrase the title to match with the study aim.
**Response:** The aim of the study must not be stated verbatim in the title and this is so in many articles in the literature.

Methodology
Study design and setting
**Comment:** The study design should be cross-sectional analytical design.
**Response:** I seem not to understand the comment. Perhaps the intended comment is that “cross-sectional” should come before “analytical” because “analytical” precedes “cross-sectional” as currently stated. That notwithstanding, the original statement has been modified in response to reviewer # 1.

Study participants and data collection
**Comment:** The author states that they selected 156 medical doctors, what was the basis for selecting this number of participants, how sure were they that by engaging 156 medical doctors the study will be able to provide reliable answer to the questions it tries to answer?
**Response:** As stated in the manuscript, convenience sampling of those that were available and disposable, was used to select the participants.

Results
Sociodemographic and background characteristics
**Comment:** When describing results, remove the word “of” after “the majority”, if there is a need of using the word “of” then it should be followed by “respondents” then number and percentage. **Response:** The sentence has been restated in line with the suggestion.

**Comment:** Table 1 should be inserted after paragraph 1 that describes sociodemographic characteristics of respondents
**Response:** The comment seems not to be clear. The table is located according to the guideline of the journal.

**Comment:** The way the total is indicated in table 1 (156) it as if 156 is the total of all the values for all the variables in the table. The best way is to indicate this total in the table title in brackets. This should be done for all the tables.
**Response:** The way the “total” is located is also conventional. However, it has been moved to the title (but without bracket)

**Comment:** In table 1, on Place of practice as a variable should have two categories i.e. Secondary health facility and Federal university teaching hospital. This will make this table easier to comprehend. Then plot a bar chart for each of these categories to indicate the distribution of specific place of practices.
**Response:** I do not think the table is difficult to understand. It is better to present the variable this way than plotting a separate graph for the subcategory.

**Comment:** To indicate less or equal use ≤ instead of <=
**Response:** The suggestion has been effected.

**Comment:** The sum of the percentages of age categories gets to 100.1, please check **Response:** This was due to approximation (rounding-up) of individual values and is acceptable. However, adjustment has been made.

Knowledge of the 2015 National Guidelines for Diagnosis and Treatment of Malaria
**Comment:** Table 2 should be cited at the end of the paragraph
**Response:** The table is located according to the guideline of the journal.

**Comments:**
- Totals should be on every major category and should be indicated in brackets, i.e. “Knowledge items (156)”, “Among those that were aware there was a national guideline for diagnosis and treatment of malaria (138)”.
- “Among those that were aware there was a national guideline for diagnosis and treatment of malaria” this can be removed and remain with “Sources of information on the national malaria treatment guideline”

**Response:** Adjustments have been made as deemed appropriate.

**Comment:** Because items in table 2 are multiple responses items, putting a 100 on the percentage as total is potentially misleading because when you sum up individual percentages goes far beyond 100.
**Response:** The total percentage has been removed.

Factors associated with good practice of the 2015 National Guidelines for Diagnosis and Treatment of Malaria
**Comment:** Why have you included all variable in your multivariable model? Usually, one would construct this using the variables with a predefined p-value from the univariable analysis. What was the criteria for inclusion of variables into the multivariable model?
**Response:** Please note that there are several methods and criteria for inclusion of variables in regression models and different scholars use different methods. The number of independent variables was modest so all were included in the model to minimize bias.

Discussion
**Comment:** Authors repeats results description in the discussion section. Kindly revise the discussion section by discussing findings rather than rewriting the findings as it appears now. **Response:** The description of results was not repeated in the discussion. Rather, the relevant/suitable results were compared with those of other studies **quantity by quantity**. That is, the **comparison** had to be **quantitative** since **quantitative studies** were being **compared**. This is a better approach because readers can easily appreciate how the results/findings compare with those of other studies quantitatively.

Conclusions
**Comment:** The authors recommend interventions that would improve attitude, it will be more meaningful to probably provide examples of such interventions.
**Response:** Interventions such as training and re-training of the medical doctors on the national guidelines could improve their attitude. This has been added to the manuscript.
