# Supplementary material for: Knowledge, attitude, and practice of the National Guidelines for Diagnosis and Treatment of Malaria among medical doctors in Ebonyi state, Nigeria: A cross-sectional survey
Source: PLoS One. 2021 Sep 20;16(9):e0257600. doi: 10.1371/journal.pone.0257600 (PMC8451992; doi:10.1371/journal.pone.0257600)
Supplement: Attachment — Submitted filename: Review comments.docx [file pone.0257600.s005.docx]

**Review comments**

**General comments**

The Author responded to the comments by the Reviewers. This is good and commendable. However, most of the responses were not reflected in the revised manuscript. Most issues as raised by Reviewers are aimed towards improving the quality of the manuscript and not necessarily for the attention of Reviewers only. This was the observation from the comments of the Author. It should be noted that there are some issues that should be attended to before the manuscript could be accepted for publication.

In line with the above thoughts, the following points should be addressed;

1. The Author commented that convenience sampling was used in the selection of the respondents. This should be included in the abstract.
2. Author should state in the manuscript the basis for inclusion of variables in multivariate analysis after bivariate analysis. This is very important as it will enable readers have a good understanding of the research.
3. The author should take another look at the grading of the outcome variables. It was stated that <50% was designated as poor, 50-75% as fair and >75% as good. The clarification needed here is why 75% should be classified as fair.
4. Like I remarked in my previous comment, the sample size for the study is 156 which is relatively low. The 95% confidence interval for the (adjusted odds ratio) apart from indicating statistical significance also gives a precision of the estimate which is a reflection of the sample size.

Based on this observation, one wonders why the Author categorized the age groups into 4 groups, professional rank and duration of service into 5 groups each. However, the Author categorized primary place of practice into 2 groups.

It is good to take a look at the 95% confidence interval in the multivariate analysis for the age group, >50 years, it is 0.1-91.3. That for >20 years of service is 0.0003-3.3.

Author should endeavor to dichotomize all independent variables in the bivariate and multivariate analysis. There should be a good explanation for categorizing any independent variable into three groups.

**Abstract**

**Methods**

The author should include how the respondents were selected

**Introduction**

Line 79, page 4, correct malarian to malaria

**Methods**

Line 92, page 4, remove the

Line 109, change the word disposable
