# Supplementary material for: Knowledge, attitude, and practice of the National Guidelines for Diagnosis and Treatment of Malaria among medical doctors in Ebonyi state, Nigeria: A cross-sectional survey
Source: PLoS One. 2021 Sep 20;16(9):e0257600. doi: 10.1371/journal.pone.0257600 (PMC8451992; doi:10.1371/journal.pone.0257600)
Supplement: Attachment — Submitted filename: Response to Reviewers_2.docx [file pone.0257600.s006.docx]

RESPONSE TO REVIEWERS

Reviewer # 1

**Comment:** Thank you for your article improvement. It is now with a good standard, to the next step. Good luck.
**Response:** Best wishes in your every endeavor.

Reviewer # 2

1. **Comment:** The Author commented that convenience sampling was used in the selection of the respondents. This should be included in the abstract.
**Response:** The suggestion has been effected.

2. **Comment:** Author should state in the manuscript the basis for inclusion of variables in multivariate analysis after bivariate analysis. This is very important as it will enable readers have a good understanding of the research. **Response:** All the independent variables were **simultaneously** entered into the multivariate regression. The number of independent variables was modest so all were included in the model to minimize bias. This has been added to the “statistical analysis” section for more clarity.

3. **Comment:** The author should take another look at the grading of the outcome variables. It was stated that <50% was designated as poor, 50-75% as fair and >75% as good. The clarification needed here is why 75% should be classified as fair.
**Response:** The author is not aware of any recommended cut-off for grading KAP of health workers. Even if “70%” or “60%” is used (instead of “75%”), the same question could still be posed as to: why 70%? Why 60? It should be noted that different authors have used different cut-offs and some categorized scores greater than 75% as good KAP or even 80% and above as good adherence. Examples:

1. Usman R. et al. Predictors of malaria Rapid Diagnostic Tests’ utilisation among healthcare workers in Zamfara State. *PLoS One*. 2018;13:12.

2. Kotepui KU et al. Knowledge, Attitude, and Practice Related to Malaria Diagnosis among Healthcare Workers in Hospitals: A Cross-Sectional Survey. *Journal of Tropical Medicine*. Volume 2019.

3. Na’uzo AM et al. Adherence to malaria rapid diagnostic test result among healthcare workers in Sokoto metropolis, Nigeria. *Malaria J*. 2020;19:2

Moreover malaria is a common illness in Nigeria, and the respondents in this study were medical doctors who are highly skilled medical practitioners that are expected to uphold optimal patient management, and this is the aim of having diagnosis and treatment guidelines. The scoring and grading approach used was consistent with the concept of the study as described under the “introduction section” of the manuscript.

4. **Comment:** Like I remarked in my previous comment, the sample size for the study is 156 which is relatively low. The 95% confidence interval for the (adjusted odds ratio) apart from indicating statistical significance also gives a precision of the estimate which is a reflection of the sample size.

Based on this observation, one wonders why the Author categorized the age groups into 4 groups, professional rank and duration of service into 5 groups each. However, the Author categorized primary place of practice into 2 groups.

It is good to take a look at the 95% confidence interval in the multivariate analysis for the age group, >50 years, it is 0.1-91.3. That for >20 years of service is 0.0003-3.3.
Author should endeavor to dichotomize all independent variables in the bivariate and multivariate analysis. There should be a good explanation for categorizing any independent variable into three groups.

**Response:** Even though the width of the confidence interval for a number of categories (>50 years of age, >20 years duration of practice) are very wide, the width of the confidence interval for most of the other categories are modest considering the relatively small sample size and dichotomizing all the variables will result in great loss of information and may also result in loss of power in detecting any difference in the overall effect. Comparing two levels of care (secondary and tertiary) makes great sense and does not indicate that other variables must be categorized into two. For instance, there is no way the professional rank will be grouped into two that it will make any great theoretical sense. There is no recommendation that all independent variables should have equal or two categories before running regression analysis. However, because of the concern over the wide confidence intervals, the “>50 years of age” and “>20 years duration of practice” categories have been merged with the preceding categories respectively to produce 3 categories of age group and 4 categories of duration of practice . It is important to note that exploratory categorization of age group and duration of practice into only two groups did not produce much changes in the width of the confidence interval compared to the current 3 categories of age group and 4 categories of duration of practice.

**Comment:** Introduction: Line 79, page 4, correct malarian to malaria
**Response:** The typo has been corrected

**Comments:** Methods
Line 92, page 4, remove the
Line 109, change the word disposable

**Response:** The typo has been corrected and “disposable” has been replaced.

Reviewer # 3

**Comments:** For the title to be more effective, it must contain the main topics of the study, in this case, the word “predictors” for knowledge, attitude and practice key in this study and it must surface in the title in any form. It is best to have the title and objective of the study marrying one another, I recommend the title be rephrased to match with the study aim.
**Response:** There are no reporting guidelines that recommend all components of the study aims and objectives must be included in the title of the study. The concept of the study did not capture “predictors” as the main topic. The purpose and basic design of the study has been captured in the title like those of many other similar articles/studies, examples:

1. Riley C et al. Knowledge and Adherence to the National Guidelines for Malaria Case Management in Pregnancy among Healthcare Providers and Drug Outlet Dispensers in Rural, Western Kenya. PLoS One. 2016;11(1): e01

2. Blanco M et al. Knowledge and practices regarding malaria and the National Treatment Guidelines among public health workers in Equatorial Guinea. Malar J. 2021;20:21

3. Na’uzo AM et al. Adherence to malaria rapid diagnostic test result among healthcare workers in Sokoto metropolis, Nigeria. *Malaria J*. 2020;19:2

4. Riley C et al. Knowledge and Adherence to the National Guidelines for Malaria Diagnosis in Pregnancy among Health-Care Providers and Drug-Outlet Dispensers in Rural Western Kenya. *Am. J. Trop. Med. Hyg.* 2018;98(5):1367–1373

5. Okoro et al. The Cross-Sectional Evaluation of the Use of Artemisinin-Based Combination Therapy for Treatment of Malaria Infection at a Tertiary Hospital in Nigeria. *Journal of Tropical Medicine*. Volume 2018.

6. Mokuolu OA et al. Provider and patient perceptions of malaria rapid diagnostic test use in Nigeria: a cross‑sectional evaluation *Malar J.* 2018;17:200

7. Akinyode AO et al. Practice of antimalarial prescription to patients with negative rapid test results and associated factors among health workers in Oyo State, Nigeria. Pan African Medical Journal. 2018; 30:229

8. Bamiselu OF et al. Adherence to malaria diagnosis and treatment guidelines among healthcare workers in Ogun State, Nigeria BMC *Public Health.* 2016;16:828

**Comment:** The author states that they selected 156 medical doctors, what was the basis for selecting this number of participants? Why didn’t they just engage 100 medical doctors? How representative is the sample to the general population of medical doctors in Ebonyi state, much as the findings from 156 medical doctors is inferred to all the medical doctors in Ebonyi state? **Response:** It is already stated in the manuscript that convenience sampling was employed in selection of participants. The 156 medical doctors were the ones that were available (during a visit to their respective places of work) and consented to participate in the study. If more or less were available and consented, more or less would have participated. Moreover, 156 is a reasonable percentage of the estimated 670 medical doctors in the Ebonyi state (according to the state chapter of the Nigerian Medical Association)
